# Cyclin/Forkhead-mediated coordination of cyclin waves: an autonomous oscillator rationalizing the quantitative model of Cdk control for budding yeast

**DOI:** 10.1038/s41540-021-00201-w

**Published:** 2021-12-13

**Authors:** Matteo Barberis

**Affiliations:** 1 Systems Biology, School of Biosciences and Medicine, Faculty of Health and Medical Sciences, University of Surrey, Guildford, UK; 2 Centre for Mathematical and Computational Biology, CMCB, University of Surrey, Guildford, UK; 3 Synthetic Systems Biology and Nuclear Organization, Swammerdam Institute for Life Sciences, University of Amsterdam, Amsterdam, The Netherlands

**Keywords:** Dynamical systems, Biochemical networks

## Abstract

Networks of interacting molecules organize topology, amount, and timing of biological functions. Systems biology concepts required to pin down ‘network motifs’ or ‘design principles’ for time-dependent processes have been developed for the cell division cycle, through integration of predictive computer modeling with quantitative experimentation. A dynamic coordination of sequential waves of cyclin-dependent kinases (cyclin/Cdk) with the transcription factors network offers insights to investigate how incompatible processes are kept separate in time during the eukaryotic cell cycle. Here this coordination is discussed for the Forkhead transcription factors in light of missing gaps in the current knowledge of cell cycle control in budding yeast. An emergent design principle is proposed where cyclin waves are synchronized by a cyclin/Cdk-mediated feed-forward regulation through the Forkhead as a transcriptional timer. This design is rationalized by the bidirectional interaction between mitotic cyclins and the Forkhead transcriptional timer, resulting in an autonomous oscillator that may be instrumental for a well-timed progression throughout the cell cycle. The regulation centered around the cyclin/Cdk–Forkhead axis can be pivotal to timely coordinate cell cycle dynamics, thereby to actuate the quantitative model of Cdk control.

## Introduction

Timing is important to living organisms: many distinct processes need to occur at definite times relative to one another, i.e. in partial synchrony and with well-defined phase differences. Examples are found in heart function, tissue differentiation, sleep/wake cycles, and adaptive responses to external challenges. Failure in the timing of processes that together establish a physiological function may compromise the viability of living cells, or may make them escape from regulation, thereby compromising the viability of multicellular organisms. And indeed, temporal coordination of the events that regulate cellular proliferation is also pivotal to health.

The eukaryotic cell division cycle is one of the clearest examples of such processes. It ensures the consecutive and alternate execution of a number of distinct and incompatible processes (‘phases’), namely cell growth (G1 phase), DNA replication (S phase), chromosome segregation (G2 phase), cell division (M phase), and in many cases cell maintenance (G0 phase). The cell would succumb and transform or develop to disease if DNA replication and cell division would occur simultaneously with multiple, possibly incomplete rounds of replication or an imbalanced DNA segregation between consecutive cell divisions.

To maintain the separation between these processes, a regulatory mechanism must be employed by the cell such that incompatible processes do appear one after the other, in a *periodic*, *unidirectional*, and *irreversible* manner. Other processes can and should partly overlap, starting at different times but ending simultaneously. Thus, the cell division cycle is a strategic choice for studying the fundamental aspects of timing, because it relies on the clearly incompatible processes of genome duplication and cell division. To determine molecular mechanisms that prevent their fatal overlap, the regulatory networks that control these incompatible processes may be explored through systems biology.

Because of its critical role in guaranteeing survival, the cell cycle network has been conserved across species during evolution, ranging from a simple, unicellular yeast to multicellular, higher organisms such as humans and plants. The cell cycle can therefore be ideally studied in model organisms such as budding yeast.

### Vital temporal coordination: keeping the incompatible separate through cyclin waves

**Fig. 1:**
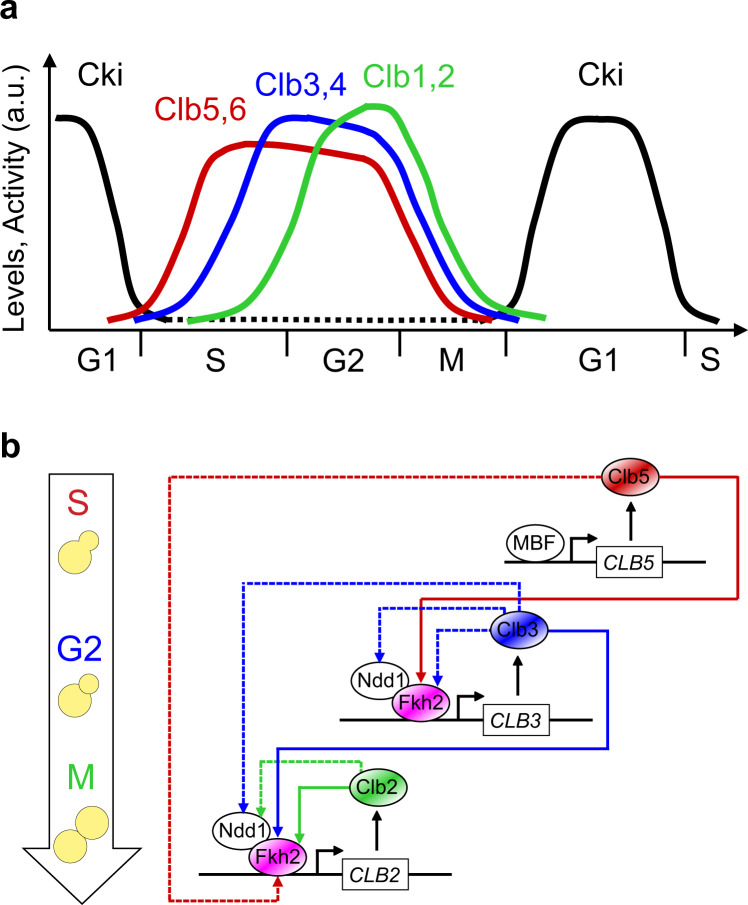
Waves of mitotic (Clb) cyclins throughout cell cycle progression. **a** Qualitative description of alternating waves of expression of mitotic cyclin/Cdk1 complexes and of their stoichiometric inhibitor Cki throughout the cell cycle phases. In budding yeast: (i) Cki indicates Sic1 (black color), which is expressed maximally in G1 phase and at a low level in the other cell cycle phases; (ii) Clb indicates mitotic cyclins: Clb5,6 (red color) trigger DNA replication in S phase; Clb3,4 (blue color) trigger completion of S phase and early mitotic events in G2 phase; Clb1,2 (green color) trigger late mitotic events and cell division in M phase. **b** Scheme of regulations connecting cyclin transcription and Clb/Cdk1 complexes through the Fkh2 transcription factor. The synchronization of Clb cyclins occurs in steps: (i) Clb5 promotes *CLB3* transcription (solid red line); (ii) Clb3 promotes *CLB2* transcription (solid blue line); (iii) Clb2 further promotes *CLB2* transcription through a Clb2-mediated positive feedback loop (solid green line) (adapted from Linke et al.^47^). Dashed colored arrows indicate regulations that may occur between Fkh2/Ndd1 and Clb cyclins, following the physical interactions that have been shown experimentally^47^. For simplicity, Cdk1 has been omitted.

The maintenance of strictly alternating cycles of genome duplication and cell division requires a regulator. Periodic waves of activity of dimeric enzymatic complexes, called cyclin-dependent kinases, represent the driving force behind cell cycle progression. These complexes are composed by a Cdk kinase – the catalytic subunit – and a differential pool of cyclins – the regulatory subunits. In the budding yeast *Saccharomyces cerevisiae*, activity of the Cdk1 kinase is modulated upon binding of nine distinct phase-specific cyclins, which are grouped in four subgroups^1–4^. Cyclins confer the substrate specificity that allows Cdk1 to drive the cell cycle through a definite order (see refs. ^5–7^ and references therein). Successive, coordinated periodic oscillations of cyclin/Cdk1 activities ensure *unidirectionality* and *timing* of cell cycle progression^8,9^: they must be activated for entry into S phase and passage through metaphase, and must be inactivated to allow cytokinesis, spindle breakdown and licensing of replication origins for new rounds of DNA synthesis. To complete these events, phase-specific cyclins are regulated to generate waves of cyclin/Cdk1 activity, a functional property of cell cycle control (Fig. 1a).

Sequential activation of cyclins by regulated transcription is crucial for the timing of cyclin/Cdk1 activities, which in turn are required for robust transcriptional oscillations by modulating the activity of various transcription factors^10–13^. Four cyclin-associated waves of transcription occur throughout the cell cycle^14–16^. *CLN1*, *CLN2*, and *CLN3* are essential for passing START at the G1/S transition^17^. *CLB5* and *CLB6* drive a timely and efficient DNA replication in S phase^18–21^. *CLB3* and *CLB4* are involved in DNA replication and mitotic spindle formation at the G2/M transition^22,23^. *CLB1* and *CLB2* are necessary for mitotic spindle elongation and mitotic exit^22–24^.

Transcriptional mechanisms regulating the expression level of cyclin waves have been widely studied^8,11–13,25^. Periodic activation of transcriptional activities normally restricted to the G1 phase occurs in cells lacking all six mitotic *CLB* genes^26,27^; however, Clb/Cdk1 activity is essential to ensure the correct timing of gene transcription in G1 phase, thus to coordinate S-G2-M events with G1 events^26^. To drive cell cycle-dependent gene expression, transcription factors must be cell cycle-regulated, and the activity of those controlling cyclin genes may be dependent on the Cdk1 activity promoted by an earlier transcriptional cyclin wave. Indeed, the cyclin/Cdk1 activity modulates transcription factors’ activity, and acts as their effector to trigger the ordered program of cyclin expression^14^. Moreover, Cdk1 and transcription network activities are coupled by feed-forward loops (FFLs) to convert periodic oscillations of Cdk activity in transcriptional response^28^, ensuring the correct temporal order of cell cycle events.

Is there a mechanism that ensures robustness of cell cycle timing? It has been shown that such mechanism exists, with cyclin/Cdk1 activities contributing to the robustness of transcriptional oscillations^29^. In fact, although Cdk1 appears not to be the main regulator of transcriptional oscillations, deletion of all mitotic, also called Clb, cyclins (*clb1*∆ *clb2*∆ *clb3*∆ *clb4*∆ *clb5*∆ *clb6*∆) results in a substantial delay of the timing of cell division^29^. It has been therefore proposed that coupling a cyclin/Cdk1 oscillator and a transcriptional oscillator may regulate cell cycle progression, such that timing of cyclin expression is controlled by the transcriptional oscillator; in turn, cyclin/Cdk1 complexes can modulate the transcriptional oscillator by controlling its amplitude and period^30–32^.

Thus, alternated waves of cyclins – thereby of cyclin/Cdk1 activities – and transcriptional events are coupled to tightly control cell cycle progression. This coupling allows to keep the incompatible processes of genome duplication and cell division separate. These incompatible processes are therefore required to begin and end in a well-defined alternating regimen.

### The cyclin/Cdk–Forkhead axis coordinates waves of mitotic cyclins

Although waves of cyclin/Cdk1 activity are critical for cell cycle transitions, it is not fully understood how the temporal occurrence of successive cyclin waves is managed. Specifically, the precise molecular circuitry responsible for the coordination of waves of cyclins is not known. As it has been highlighted in the fundamental contribution to the cell cycle field written in 1998 by Mendenhall and Hodge, the transcriptional regulation of a number of cyclin genes is not known^33^.

Efforts have been made to identify the transcriptional network governing phase-specific waves of gene expression, both experimentally (see refs. ^12,13^ and references therein) and computationally (see refs. ^27,34–37^ for some of the many developed methodologies). For example, Clb5 and Clb2, the more abundant cyclins within the Clb5/Clb6 and Clb1/Clb2 pairs^38^, respectively, are known to be activated through transcriptional heterodimers: the former by the Mlu1 cell cycle box (MCB)-binding factor, MBF (Mbp1/Swi6 dimer); the latter by the Swi-five factor, SFF (Mcm1/Fkh2/Ndd1 trimer)^12,13^. The mechanism through which transcription of these mitotic *CLB* cyclin genes is controlled involves Cdk activities^8,11,39^, however it lacks a comprehensive understanding.

Clb2/Cdk1 activity, main regulator of the timing of cell division, promotes transcription of the *CLB2* gene itself by a positive feedback loop (PFL). The regulation occurs through phosphorylation of the Forkhead (Fkh) transcription factor Fkh2^8^ which, together with its cognate Fkh1, promotes cell division by regulating the *CLB2* cluster that drives the G2/M gene expression^40–43^. Of note, this leads to the paradox that cell division would not initiate in absence of Clb2. Furthermore, *CLB2* transcription is not induced in the absence of Clb1-4-associated kinase activities^8^ and Clb5/Cdk1 can phosphorylate Fkh2^44^, suggesting Clb5 as the trigger of *CLB2* transcription. Strikingly, Fkh2 has been shown to be phosphorylated in vivo in a cell cycle-dependent manner during the G2/M transition, therefore suggesting the potential involvement of other mitotic Clb/Cdk1 activities, beside Clb2/Cdk1^40^, which may be important for the accumulation of Clb1 and Clb2^45^.

Thus, it is not known whether or not Clb/Cdk1 activities – with the exception of Clb2/Cdk1 – are required for *CLB2* transcription, and whether or not Clb/Cdk1 complexes directly regulate *CLB2* promoter or make use of additional mechanisms – as pointed out earlier^46^. In addition, already in 1998 Mendenhall and Hodge stressed that, among the missing details of the cyclin/Cdk1-mediated transcription: ‘*Not all of the dominoes have been identified; virtually nothing is known about the factors regulating CLB3 and CLB4 transcription, for example. Completing the identification and the characterization of the interrelationships among these factors remains a major challenge in this field*’^33^. Altogether, identification of the interdependencies between Fkh and cyclin-associated kinase activities for a timely *CLB2* expression, as well as of the ‘*factors regulating CLB3 and CLB4 transcription*’^33^ has therefore been a major challenge in the cell cycle field since the last two decades.

To shed new light on the dynamic coupling between Fkh and cyclin-associated Cdk1 activities, the former have been recently investigated as targets for the latter. Through a systems biology-driven investigation of the interconnection between these molecular players responsible for the timely coordination of DNA replication with cell division, the sequential order of waves of Clb cyclins was demonstrated to be achieved by mutual coordination of Clb/Cdk1 activities with Fkh-mediated transcriptional activity^47^ (Fig. 1b).

In detail, a minimal mathematical, kinetic model of Clb/Cdk1 activities – implemented through Ordinary Differential Equations (ODEs) – was generated that predicts a Clb/Cdk1-mediated regulation of an activator molecule responsible for the control of *CLB3* transcription. This prediction was successfully validated experimentally, through identification of Fkh2 as pivotal molecule: Clb cyclin waves are synchronized by Fkh2, and a Clb/Cdk1-mediated regulation of Fkh2 modulates Clb cyclin expression through a FFL^47^. Thus Clb/Cdk1 and Fkh2 mutually coordinate one another. Fkh2 specifically binds to the *CLB3* promoter, and promotes *CLB3* expression as well as the timely appearance of Clb3 protein level. Beside the known interactions with Clb2 (M-phase cyclin) and Clb5 (S-phase cyclin), Fkh2: (i) stably interacts with Clb3 (G2-phase cyclin)^47^; (ii) co-localizes with Clb3, but not with Clb2, in S phase^47^; and (iii) is phosphorylated by Clb3/Cdk1^47^, besides by Clb2/Cdk1 and Clb5/Cdk1^8,40,44,46,48^. In addition, Fkh2 has been shown to affect the formation of the mitotic spindle^40^ – where also Clb5 is involved^49,50^ –, thus suggesting that it might regulate Clb3 that is involved in this process^51^.

Of note, accumulation of *CLB4* but not *CLB3* transcript levels are affected by Fkh1 deletion^47^, in addition to early studies that showed Fkh1 binding to the *CLB4* promoter^16^, suggesting a potential involvement of Fkh1 in *CLB4* gene repression. This result calls for a different transcriptional mechanism regulating *CLB3* and *CLB4* expression^47^.

A design principle, i.e. a definite topology underlying a network that can robustly achieve a particular biological function, emerges in which sequential waves of *CLB* cyclins transcription and Fkh2 activity mutually coordinate, with Fkh2 acting as a transcriptional timer to modulate mitotic Clb/Cdk1 activities through a Clb/Cdk1-mediated feed-forward regulation. This synchronization of mitotic Clb cyclins provides a rationale for their sequential appearance, by temporally coordinating the expression of *CLB* waves, and is realized by the bidirectional interaction between Clb cyclins and Fkh2, resulting in a well-timed progression throughout the cell cycle (Fig. 1b). This design has been further supported by in silico simulations through a minimal Boolean-based model of Clb/Cdk1 kinase activities, which produces robust, cyclic oscillations of the mitotic Clb states compatible with the involvement of an activator molecule – the Fkh2 transcription factor – in a linear, progressive activation of *CLB* cyclins^47^.

**Fig. 2:**
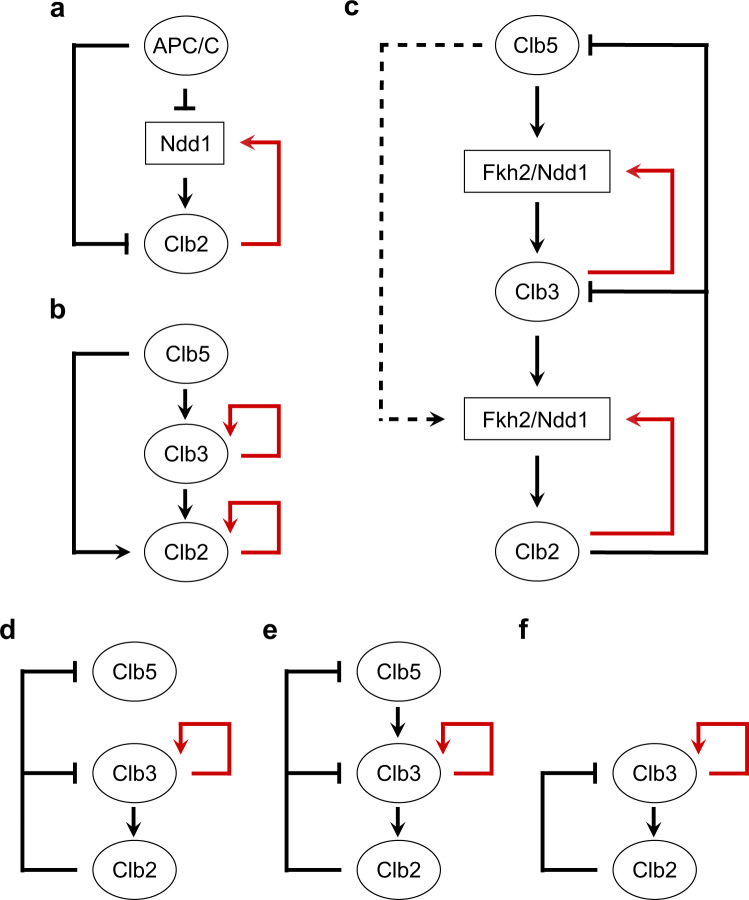
Design principle underlying the minimal model of cell cycle control in budding yeast. **a** Coherent type II feed-forward loop (FFL, black lines) generated among the Anaphase-Promoting Complex/Cyclosome activated by Cdh1 (APC/C^Cdh1^), Ndd1 and their target Clb2 (adapted from^54^). **b** Coherent type I FFL (black arrows) proposed to occur among the mitotic Clb cyclins: Clb5 activates Clb3, and Clb5 activates Clb2 together with Clb3. **c** Detailed regulations occurring within the coherent type I FFL in **b**, through the Fkh2/Ndd1 transcriptional complex. The Clb5 → Clb2 regulatory activation is shown in dotted arrow, to indicate its possible less relevance within the FFL. Clb2-mediated inhibitory regulations of Clb3 and Clb5 (through APC/C, not visualized) identified in autonomous Clb/Cdk1 oscillators are indicated by bar-headed black lines. **d**, **e** Most frequently dominant regulations underlying autonomous oscillations^85,86^. **d** Two Fkh2-mediated activatory regulations (Clb3 → Clb2 and Clb3 PFL) and two APC/C-mediated regulatory inhibitions (Clb3 ⊦ Clb2 and Clb5 ⊦ Clb2). **e** Three Fkh2-mediated activatory regulations (Clb5 → Clb3, Clb3 → Clb2 and Clb3 PFL) and two APC/C-mediated regulatory inhibitions (Clb3 ⊦ Clb2 and Clb5 ⊦ Clb2). **f** The ‘negative feedback with positive feedback loop’ (NF-PFL) design: two Fkh2-mediated activatory regulations (Clb3 → Clb2 and Clb3 PFL) and one APC/C-mediated regulatory inhibition (Clb3 ⊦ Clb2). **a–e** For clarity, Clb cyclins and APC/C that drive post-transcriptional regulations are indicated with ovals, whereas Fkh2 and Ndd1 that promote transcriptional regulations are indicated within squares. Bar-headed black lines indicate APC/C-mediated inhibitory reactions, whereas red arrows indicate positive feedback loops (PFLs). For simplicity, Cdk1 has been omitted.

Fkh proteins are bound to promoters throughout the cell cycle, and their periodic activity is dependent on cell cycle-regulated recruitment of the coactivator Ndd1 at the S/G2 transition^52,53^. Similarly to Clb/Cdk1 complexes, Ndd1 also exhibits dynamics of activation and deactivation: Ndd1 degradation, through the Anaphase-Promoting Complex/Cyclosome activated by Cdh1 (APC/C^Cdh1^), generates a feed-forward regulation that governs the timing of its accumulation at the G1/S transition^54^ (Fig. 2a).

Furthermore, similarly to Fkh2, Ndd1 activity is dependent by kinase activity, specifically of Clb5/Cdk1^44^, Clb2/Cdk1^46^, and Cdc5^55^. Thus, multiple mitotic Clb/Cdk1 complexes may be able to phosphorylate Ndd1 to promote its association to Fkh2 at the *CLB2* promoter. The recent results support this view, with (i) Ndd1 stably interacting with Clb3, (ii) the Fkh2/Ndd1 complex co-localizing with Clb3 in S phase; and (iii) Ndd1 (and Fkh2) being strongly enriched to the *CLB3* promoter^47^. These findings, together with the evidence that Ndd1 is phosphorylated by Clb3/Cdk1^7^, indicate that the association between Ndd1 and Fkh2 oscillates throughout the cell cycle and correlates temporally with the transcriptional activation of *CLB3* and *CLB2*.

Altogether, a novel design principle is uncovered, where Clb/Cdk1 kinases and Fkh2/Ndd1 transcription activities are interlocked to control gene regulation. Within this tie, the Fkh2/Ndd1 transcriptional complex may be regulated by multiple Clb/Cdk1 activities, to guarantee a timely cell division.

### Network motifs underlying the Fkh2/Clb3 axis

The emergent properties of cell division can be investigated and reproduced by modeling efforts, and minimal mathematical models have been recently developed to capture essential behaviors of cell cycle temporal dynamics^47,56–59^. These models were inspired by the experimental evidence that cells carrying a single mitotic cyclin/Cdk complex are progressing through the cell cycle in mouse^60^ and fission yeast^61^.

Through simulation of a minimal model of the mitotic Clb/Cdk1 regulation in budding yeast, the computer-based prediction of the transcriptional regulation embedded in the linear cascade of Clb cyclin activation – Clb5,6/Cdk1 activate Clb3,4/Cdk1, which in turn activate Clb1,2/Cdk1 (Clb5 → Clb3 → Clb2) – has been validated experimentally^47^ (Fig. 1b). The Fkh2 transcription factor was uncovered to control the temporal expression of mitotic *CLB* waves, with its activity being modulated by Clb/Cdk1 complexes throughout the cell cycle^47^. This transcriptional regulation may be realized with Clb5/Cdk1 and Clb3/Cdk1 promoting *CLB2* transcription by phosphorylating Fkh2, either through the transcriptionally mediated linear Clb cascade (Clb5 → Clb3 → Clb2), or through a FFL in which the linear cascade can play the main role (Fig. 2b). FFLs are ‘network motifs’ highly favored during the evolution of transcriptional regulatory networks in budding yeast^62,63^, and are described by three genes coding for transcription factors, say *X*, *Y*, and *Z*, with *X* and *Y* directly regulating *Z* and *X* regulating *Y*^64^. The three regulations that occur among these three components can be activatory or inhibitory.

In an ‘incoherent’ FFL, the signs of the direct regulation – from *X* to *Z* – is opposite than the overall sign of the indirect regulation – from *X* to *Z* through *Y*. The ‘incoherent’ FFL generates pulses and accelerates or delays responses and, for this reason, it is referred to as ‘sign-sensitive accelerator’^65^. In a ‘coherent’ FFL, the sign of the direct regulation – from *X* to *Z* – is the same as the overall sign of the indirect regulation – from *X* to *Z* through *Y*. The ‘coherent’ FFL may serve as a sign-sensitive delay element: a short pulse of *X* or *Y* is not sufficient to activate *Z* and, for this reason, this network motif is referred to as ‘persistence detector’^64,66^.

Incoherent and coherent FFLs have been shown to convert periodic cyclin/Cdk1 activity to transcriptional response in budding yeast, through direct phosphorylation of both executor proteins and the transcription factors that regulate their expression^28^. Furthermore, a ‘coherent type II’ FFL^67–69^ is in place between the APC/C^Cdh1^, the Ndd1 transcription factor and their common targets Clb2 (*CLB2* transcription), to control the dynamics of Ndd1 activation throughout cell cycle progression (Fig. 2a). This FFL involves both transcriptional and post-translational regulations^54^. Therefore, cyclin/Cdk1 complexes drive progression throughout successive cell cycle phases through FFLs-mediated networks, thus guaranteeing alternation between the incompatible processes of initiation of DNA synthesis and cell division.

Taking into account the early evidence^44^ and the most recent work^47^, it may be hypothesized that a FFL is the design principle that governs oscillatory waves of Clb cyclins. Specifically, a ‘coherent type I’ FFL can be in place, where Clb5 (X) positively regulates Clb3 (Y), with both jointly regulating Clb2 (Z). Thus, the FFL involves Clb5 → Clb3 (X → Y), Clb3 → Clb2 (Y → Z), i.e. the linear cascade Clb5 → Clb3 → Clb2 that indirectly connects Clb5 and Clb2, and the direct connection Clb5 → Clb2 (X → Z) (Fig. 2b).

Importantly, it is at present not known whether a phosphorylation threshold of the Fkh2 transcription factor, mediated by adequate levels/activities of Clb5/Cdk1 and Clb3/Cdk1 complexes, needs to be reached to promote *CLB2* transcription. In this context, PFLs – where dynamics of a component *X* are positively influenced by itself – may assist in reaching a possible required threshold of Fkh2 activation. Indeed, PFLs hold a relevant role in signal amplification, such for example: (i) the auto-activatory process in which Clb2/Cdk1 phosphorylates Ndd1 to promote *CLB2* transcription^54^ (Fig. 2a, red arrow); and (ii) the auto-activatory processes in which Clb2/Cdk1 phosphorylates Fkh2 to promote *CLB2* transcription^8^ and Clb3/Cdk1 phosphorylates Fkh2 to promote *CLB3* transcription^47^ (Fig. 2b, red arrows), both through the Fkh2/Ndd1 transcriptional complex (Fig. 2c). Interestingly, a Clb3/Cdk1-mediated PFL on *CLB3* transcription (Clb3 → Clb3, Y → Y) may contribute to generate the Clb oscillatory pattern together with the direct regulation of Clb3/Cdk1 on *CLB2* transcription (Clb3 → Clb2, Y → Z)^47^.

PFLs enhance amplitude and robustness of cyclin/Cdk oscillations in the mammalian cell cycle^70–72^, also in combination with negative feedback loops (NFLs)^72^. In budding yeast, PFLs and NFLs have been shown to keep the coherence of entrance in or exit from cell cycle transitions. Cln1,2/Cdk1- and Clb1,2/Cdk1-mediated PFLs regulate G1/S and G2/M, respectively^5,8,73^, whereas Clb1,2/Cdk1-mediated NFLs regulate M/G1 through activation of the APC/C^Cdc20^- and APC/C^Cdh1^-mediated abolishment of waves of Clb activities (see^56^ and references therein). Although the Clb3/Cdk1-mediated PFL (Clb3 PFL) has the ability to timely shape certain Clb waves in silico^47^, its relevance for the Clb dynamics in vivo has yet to be further investigated.

Systems biology research has revealed a mechanism in which Clb/Cdk1 activity and transcription trigger cell cycle progression, with a progressive Fkh2 activation that may be realized by multiple Clb/Cdk1 complexes. This regulatory mechanism may be realized through a linear cascade (Clb5 → Clb3 → Clb2) or through a coherent FFL in which the linear cascade has possibly a major role, either aided by Clb2/Cdk1-mediated – and, ideally, Clb3/Cdk1-mediated – PFLs. Interestingly, it has been speculated that combining a coherent FFL and a PFL could lead to a committed transition^74^.

### The Fkh2/Clb3-mediated regulations underlie autonomous oscillations of cyclin waves

Natural cell cycle oscillations are characterized by incoherent FFLs, such those observed in the cell cycle of early *Xenopus laevis* embryos^75^. Incoherent FFLs can: (i) exhibit biphasic responses with regard to time or dose^76^, (ii) distinguish between oscillatory and sustained signals with proper network parameters^77^, and (iii) enhance network robustness^78^. Conversely, coherent FFLs are found in gene regulatory and signaling network, and sustain – with both positive and negative inputs – network robustness against perturbations^79^. This feature may allow for a rapid response and sustained oscillations^79^. Furthermore, among the network designs that have been described to characterize cell cycle oscillators, PFLs promote oscillations and switch-like responses that allow unidirectionality of cell cycle progression, enhancing amplitude, and robustness of cyclin/Cdk oscillations^70–72^.

In the budding yeast cell cycle, both cyclin/Cdk1-mediated incoherent and coherent FFLs have been observed, where periodic fluctuations in the Cdk1 activity involve the Cdk1-mediated phosphorylation of a substrate executor protein and its transcription factor^28^. Incoherent FFLs can drive cell cycle control^80^; however, NFLs, but not incoherent FFLs, exhibit robustness to changes in stimulus duration in response to oscillatory stimulation^81^.

Recently, the role of the Fkh2 transcription factor as temporal coordinator of mitotic Clb waves was uncovered, identifying Fkh2 as a molecule controlling the sequential activation of *CLB* expression^47^. Although *CLB2* activation through Fkh2 phosphorylation may occur either through a linear cascade (Clb5 → Clb3 → Clb2) or through a coherent FFL, in which the linear cascade has possibly a major role (Fig. 2c), coherent FFLs or a more sophisticated motif that incorporates together FFL + PFLs have not been shown yet to promote cyclin/Cdk1 oscillations in budding yeast. Noteworthy, the linear cascade (Clb5 → Clb3 → Clb2) is able to exhibit cyclic Clb oscillations in a Boolean type of modeling effort^47^.

This result did stimulate a deeper investigation about the designs responsible for autonomous cell cycle oscillations in budding yeast. Specifically, the number and nature of sets of motifs that were able to synchronize the oscillatory-like behavior of Clb/Cdk1 waves, thus reflecting the alternation of DNA replication and cell division, have been explored. Practically, network motifs that characterize autonomous oscillators were investigated by applying the System Design Space (SDS) methodology^82–84^. This methodology relates genotype (in terms of the parameter values) to phenotype (referring to the combination of dominant reactions that may define a particular trait) by deconstructing a biochemical system into a finite number of qualitatively distinct network structures. Computationally, this translates into the following: for a given set of parameters and concentrations of model species, there exists a single ‘dominant positive term’, i.e. largest, and a single ‘dominant negative term’ in each ordinary differential equation (ODE). When reducing the mathematical description of the phenotypes to these dominant reactions, boundaries in the parameter space where the phenotype is valid may be obtained, and phenotypes may be sampled for oscillations^84^.

This methodology has been employed to explore the areas in the full parameter and reaction state space where particular network structures (phenotypes) are prevalent (dominant) in the experimentally validated minimal model of Clb/Cdk1 regulation (see Linke et al.^47^ and reference therein). Through this analysis, which was improved by adding a search for complex conjugate eigenvalues with positive real part expected around Hopf bifurcations – from which limit cycles may arise – a search was conducted to explore whether, and to which extent, alternative motifs exist that contribute to the temporal and sustained coordination of Clb/Cdk1 complexes^85^. Autonomous oscillations (also referred to as ‘limit cycles’) capturing the sequential activation and inactivation of waves of the three mitotic Clb/Cdk1 complexes and their stoichiometric inhibitor – Sic1 (G1 phase), Clb5,6/Cdk1 (S phase), Clb3,4/Cdk1 (G2 phase), and Clb1,2/Cdk1 (M phase) – were observed. A definite regulatory mechanism was identified that incorporates Clb3/Cdk1-centered regulations that self-sustain Clb/Cdk1 and Sic1 oscillations: a Clb3/Cdk1-mediated PFL, and the linear cascade of activation of mitotic Clb/Cdk1 complexes through Clb3/Cdk1^85,86^ (Fig. 2d, e).

Specifically, the activatory regulations Clb5 → Clb3 and Clb3 → Clb2, forming the recently discovered Fkh2-mediated linear *CLB* cascade^47^ (Fig. 2d, e, solid black arrows), were found to be more frequently dominant in network designs that yield autonomous Clb/Cdk1 oscillations as compared to the Clb5 → Clb2 regulation described earlier^44^ (Fig. 2c, dotted black line). Moreover, a PFL mediated by Clb3/Cdk1 on *CLB3* synthesis (Clb3 PFL) – or by Clb2/Cdk1 on *CLB2* synthesis (Clb2 PFL) in absence of Clb3 PFL – improved the ability of the minimal model to generate sustained Clb/Cdk1 oscillations^85^ (Fig. 2d, e, solid red arrows), in agreement with early in silico analyses that predicted the ability of the Clb3 PFL to timely shape certain Clb waves^47^, and with the hypothesis that the FFL + PFLs structure underlies a well-timed cell division.

Furthermore, with respect to the inhibitory regulations, the Clb2/Cdk1-mediated APC/C NFLs on Clb5/Cdk1 and Clb3/Cdk1 were more frequently dominant in network designs that yielded autonomous Clb/Cdk1 oscillations^85,86^ (Fig. 2d, e, bar-headed black lines).

In summary, in budding yeast, the Fkh2/Clb3 axis underlies autonomous oscillations of Clb/Cdk1 activities^85^ and their mutual coordination with Fkh2 transcriptional activity^47^. Of note, a design in common between the network structures that exhibit oscillations can be observed: two activatory regulations through Fkh2 (Clb3 → Clb2 and Clb3 → Clb3) and one inhibitory regulation through APC/C (Clb3 ⊦ Clb2) (Fig. 2f). A PFL mediated by Clb3/Cdk1 on *CLB3* synthesis (Clb3 PFL), improves the ability of the models to generate sustained Clb/Cdk1 autonomous oscillations. This design has been recently named ‘negative feedback with positive feedback loop’ (NF-PFL)^86^, and is a robust oscillator^87^. Because Fkh2 is conserved across yeast species, including fission yeast^88^ and *Candida albicans*^89^, and in filamentous fungi^90^, it can be speculated that a network where Fkh2 modulates, and is modulated by cyclin/Cdk complexes may be involved in the order in time of incompatible cell cycle processes.

### Outlook 1: CHECKPOINT versus AUTONOMOUS OSCILLATOR models of cell cycle control

Time-dependent responses of biological networks, such those occurring in the cell division cycle, may be accompanied by oscillatory behavior of their components, to convert stimuli to physiological output at a proper timing. A deregulation of this timing, thus of the staggered cyclin/Cdk oscillations that respond to extra- and intra-cellular signals, may impact on the coordination of the incompatible processes of DNA replication and cell division. Therefore, network designs (motifs) that exhibit timely oscillations are inherently crucial to sustain organismal physiology.

In budding yeast, cell cycle networks are typically modeled through the CHECKPOINT logic, which explicitly considers irreversible transitions between cell cycle states. In these models, developed by Tyson and Novák, the starting point of the simulations is reset upon reaching specific concentration thresholds of Clb5 for the onset into S phase, and for Clb2 for the onset into G2/M phase^91,92^. Experimentally, these simulations correspond to scenarios where DNA damage/errors would activate the checkpoint affecting Clb5 levels – thus slowing/halting DNA replication dynamics – and where troubles in completing mitotic events would activate the checkpoint affecting Clb2 levels – thus delaying/impairing cell division. Therefore, in these models, oscillations may not be autonomous. Network motifs such as PFLs or NFLs – and their combinations – are able to generate oscillations in budding yeast^93^, and the cell cycle may have been designed by evolution to oscillate, e.g. when no checkpoint needs to be activated. Therefore, it is remarkable that the AUTONOMOUS OSCILLATOR logic has not been shown yet for the available wild type yeast CHECKPOINT models.

There may seem to be a fine line between the CHECKPOINT and AUTONOMOUS OSCILLATOR views, which may have resulted in a conceptual misinterpretation for more than two decades. A few published cell cycle models may look as if they are autonomously oscillating; however, looking at how they have been implemented mathematically, they are not. Two examples of the CHECKPOINT logic considered by Tyson/Novák may be examined where seemingly autonomous oscillations are shown for a cell cycle model of fission yeast (indicated below as the 1997 model)^94^ and for a generic model of eukaryotic cell cycle regulation (indicated below as the 2006 model)^95^, differently from the AUTONOMOUS OSCILLATOR logic considered in cell cycle models by Goldbeter in mammalian cells^96–98^, by Ferrell in *Xenopus laevis*^99–101^, and by Barberis in budding yeast^85^, where autonomous oscillations are found for minimal to medium-size models of the cyclin/Cdk network.

The 1997 model does contain checkpoints of the following type: (i) when *SPF* crosses 0.1 from below, S phase is initiated (Start); (ii) when *UbE* crosses 0.1 from above, the cell divides functionally (i.e. mass is divided by 2); and (iii) 60 min after Start, *k*_*p*_ is divided by 2, and at cell division *k*^*p*^ is multiplied by 2 (see Table 1 of that work for details). These rules indicate that, while the model is running, there are points in model time where variables are reset (e.g. mass, which directly affects rates in the system) or where parameter values are changed (k_p_). This means that, as the model is running it is forced by the events to jump non-continuously through the state space and parameter space. This is a significant difference compared to autonomous oscillatory models, among which the minimal model of the mitotic cyclin/Cdk1 network of budding yeast discussed here (indicated below as the 2020 model)^85^, which contain neither of these jumps, and do not reset. As a consequence of such parameter and state space jumps and enforced oscillations in mass and parameters, in the 1997 model^94^ an increased likelihood of observing oscillations (either transient or permanent) exists. Similarly to the 1997 model, the 2006 model^95^ uses the same implementation of the mass variable and mass-dependent checkpoints, i.e. division (*mass* = *mass*/2), when *actCycB* decreases to 0.1 (fission yeast), 0.2 (budding yeast), and 0.3 (mammalian cell).

A difference between the 1997 and 2006 models by Tyson/Novák is that the 2006 model only uses a checkpoint that affects a single variable (mass). The mass then indirectly affects the other model variables by adjusting rates in the system, but it does not alter parameters during model runtime as it instead occurs in the 1997 model. In that sense, the 2006 model is closer to the approach shown for the 2020 model by Barberis, but still not quite the same because there are enforced state space jumps which do not occur in the latter. An interesting way of thinking about the difference between the 2006 and 2020 models is that, in the 2020 model, once the model starts to evolve in time and relaxes to a limit cycle oscillator it will permanently remain in the path determined by the limit cycle. Conversely, in the 2006 model, at specific points during the time course when the division event is triggered, the model is forced out from its current state into another state (*mass* = *mass*/2). This new state may or may not be in the attractor region where it was before. Of note, the steady state stability properties should remain the same, since no parameters were changed. In the 1997 model, the jump is even more severe due to the parameter changes that are performed, which may induce changes in the presence of, and stability of steady states, e.g. it may induce a bifurcation. The approaches shown for the 1997 and 2006 models are interesting from a computational point of view, but different from the approach considered in the 2020 model.

Of note, in the 2006 paper, it is reported that the bifurcation points, including the important SNIPER, occurs at a fixed mass. In all checkpoint models of the cell cycle, bifurcation analysis is performed by fixing the mass variable and turning it into a parameter. As a consequence, the model being integrated is then different than a model where the mass is a dynamic variable. This can also be observed in the 1997 model. Under such circumstances, the CHECKPOINT logic may reduce to something similar to the AUTONOMOUS OSCILLATOR logic of the 2020 model, given that there are no other checkpoints left in the model. Importantly, the 2020 model structure was not designed to yield oscillations in general, but it can yield oscillations. Specifically, the 2020 model shows that Clb3-centered interactions are the network motifs underlying mechanisms of these oscillations^85^ that have been proven to exist in budding yeast cells^47^.

The prediction that Clb3-centered regulations are the highest represented network motifs that drive autonomous oscillations in a minimal model of cyclin/Cdk control^85^ provides a possible ground to reconcile CHECKPOINT and AUTONOMOUS OSCILLATORY views. In a view of a dynamic cell cycle, autonomous oscillations driven by Clb3, thus by the Clb3/Cdk1 kinase complex, may occur when a coupling to the S and M phase kinase complexes is realized through a series of ‘*clocks*’ which coordinate together: Clb5 (CLOCK1), Clb3 (CLOCK2), and Clb2 (CLOCK3)^102^. A recent mechanism has been proposed where ‘*clock unit*s’ control waves of Clb activities, and therewith the temporal coordination of Clb/Cdk1 complexes: CLOCKS (Clb cyclins), DRIVER (Cdk1 kinase), TIMER (Sic1 inhibitor), CONTROLLER (Fkh2 transcription factor), and MODULATOR (Sir2, histone deacetylase)^102^. Within these ‘*clock units*’, Clb5 and Clb2 respond to the checkpoint mechanisms (Tyson/Novák view), and Clb3 drives autonomous cell cycle oscillations coordinating Cb5 and Clb2 (Barberis view) to maintain cell proliferation. Thus, being Clb3 tightly coordinated with Clb5 and Clb2, an autonomous oscillator may be maintained (through Clb3-centered regulations) until the action of a checkpoint (through Clb5 and/or Clb2), which activation would then terminate the autonomous oscillations. This view provides a possible solution to the conceptual misinterpretation between CHECKPOINT and AUTONOMOUS OSCILLATORY logics, reconciling these views.

**Fig. 3:**
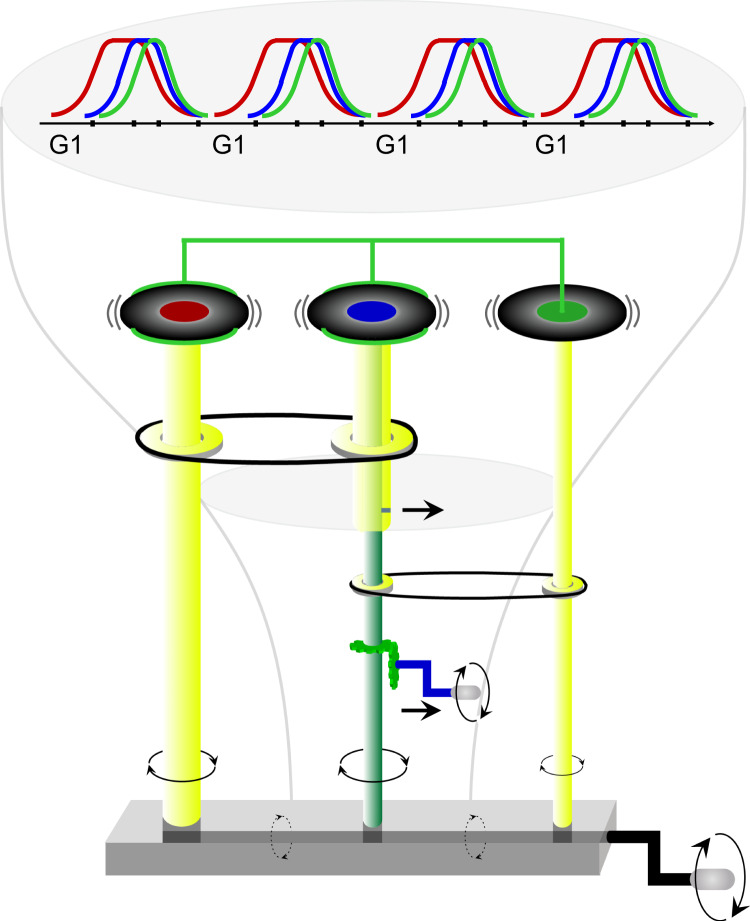
Gear shifter mechanism of Clb-centered cell cycle control. An autonomous pattern of alternating waves of mitotic cyclins over time may occur through a gear shifter mechanism. Clb cyclins ‘read’ the cell’s state and the Cdk1 kinase ‘writes’ that state by modulating its activity. The ‘readers’ (Clb)–centered gear shifter is realized through a scaffold formed by three schafts (yellow vertical pipes) each corresponding to the main mitotic Clb cyclins: Clb5 (red disc), Clb3 (blue disc), and Clb2 (green disc). Of note, a yellow and a green vertical pipes are connected through a catch-and-release mechanism (little horizontal black line), which allows the two pipes to move together. When the gear shifter is actioned (through the black crank), movement of Clb5 activates the coordinates motion of Clb3 and, in turn, of Clb2 through two black bands, thus resembling the two Fkh2-mediated regulations occurring within the linear *CLB* cascade, Clb5 → Clb3 and Clb3 → Clb2 (black arrows in Fig. 2e). When movement of Clb3 is required to be further expedite, the catch-and-release mechanism connecting the yellow and green vertical pipes of Clb3 is removed from a fixed position (through the blue crank that sets in motion the green cogs). This step allows the green pipe to function independently from the yellow pipe and to further boost Clb2 movement (through the black band connecting Clb3 and Clb2), thus resembling the activation of the PFL mediated by Clb3/Cdk1 on *CLB3* synthesis (Clb3 PFL) (red arrow in Fig. 2e). The gear shifter mechanism also includes a brake system connecting the three Clb disks that is activated by the move of Clb2, which progressively reduces the move of Clb3 and of Clb5, thus resembling the two APC/C-mediated regulatory inhibitions, Clb3 ⊦ Clb2 and Clb5 ⊦ Clb2 (bar-headed black lines in Fig. 2e) driven by Clb2. Altogether, modulation of the gear shifter mechanism on Clb3 allows for the maintenance of an autonomous coordination of the three Clb disks.

Clb cyclins, thereby Clb/Cdk1 activities, may be coordinated through a ‘reader-writer’ mechanism proposed for enzymatic gear shifters that exhibit a double functionality, one by which they ‘read’ the cell’s state and one by which they ‘write’ and modulate that state^103^. In this sense, Cdk1 ‘writes’ by phosphorylating target proteins upon the binding of ‘readers’ Clb cyclins, or ‘*clock units*’, that activate Cdk1 by determining which of the possible protein substrates it will phosphorylate, thus determining Cdk1 specificity. ‘Readers’ and ‘writer’ operate in a quasi-independent manner in real time: as the cell cycle is running, Cdk1 first associates with one, then with a second, and then with the subsequent cyclins, to generate the characteristic waves of cyclins pattern over time. Within this scenario, the CHECKPOINT and AUTONOMOUS OSCILLATORY logics are reconciled through a ‘readers’–centered gear shifter mechanism (see Fig. 3 and its description in the accompanying figure legend) that sets in motion the activatory (Clb/Fkh2-mediated) and inhibitory (APC/C-mediated) regulations coordinating Clb waves (Fig. 2e).

After three decades from the pioneer in silico studies of Goldbeter and Tyson, who did show that a progressive activation and inactivation of a single cyclin/Cdk complex is able to generate its sustained oscillations^104,105^, a minimal autonomous cell cycle oscillator independent of checkpoints mechanisms is discovered – for the first time – for budding yeast. It cycles by itself without any periodic reset, exhibiting sustained oscillations of mitotic Clb/Cdk1 complexes through a progressive accumulation of cyclin levels – thereby progressive activation of cyclin/Cdk complexes from S-to-M phase – to ensure *unidirectionality* of cell cycle progression. In principle, this scenario reflects the logic of the quantitative model of Cdk control that has been envisioned by the Nobel Prize 2001 recipient Sir Paul Nurse in 1996. This model proposes that a progressive cyclin accumulation leads to an increase in the Cdk activity through different thresholds of activity, with different thresholds of cyclin-mediated Cdk activity dictating progression through S phase and M phase^106,107^. The molecularity underlying Sir Nurse’s quantitative model of Cdk control is currently not fully revealed, and the novel design principle proposed here may fill this gap in the knowledge for budding yeast, through exploration of the recently proposed ‘*clock unit*’ mechanism underlying the waves of cyclins pattern, where a progressive Fkh2 activation may be realized by the action of multiple Clb/Cdk1 complexes^102^.

### Outlook 2: Role of cyclins for a well-timed cell cycle progression

Because well-timed DNA replication and cell division maintain a healthy offspring, timely functioning of events driven by cyclins is ensured by their partially overlapping activities^5^. For example, this overlap guarantees DNA replication to take place at a correct timing^108^. Clb6 and Clb5 are partially overlapping in the regulation of early and late DNA replication, respectively, with Clb5 being the main regulator of the process, replacing Clb6 function in *clb6*∆ cells. In *clb5*∆ cells, S phase is prolonged^21^, and the correct replication timing may be restored progressively after Clb2 activation^108^. Conversely, in *clb2*∆ cells, defects in mitotic entry and delay in mitotic exit are observed^23^ because Clb5 or Clb3 cannot replace the missing Clb2 activity. Differently from Clb5 and Clb2, which deletions impact on the cell division timing, Clb3 deletion does not affect cell cycle timing^109^. In fact, in *clb3*∆ cells, cell division occurs at a correct timing because Clb2 can replace Clb3 activity. However, Clb3 deletion leads to an altered dynamic of cell division and is lethal in the *clb2*Δ *clb3*Δ double mutant^23^, and in the *clb5*Δ *clb3*Δ *clb4*Δ^19^ and *clb2*Δ *clb3*Δ *clb4*Δ^22,23,110,111^ triple mutants. In these scenarios, Clb5 and Clb2 replace Clb3 (and Clb4) activity required for mitotic events such as spindle formation. Altogether, this bulk of evidence indicate that overlapping of waves of cyclins is instrumental to guarantee a correct timing of cell division.

Interestingly, Clb3 appears to be not evolutionarily conserved. Yet, this potential, yet uncovered, function of this mitotic cyclin highlights that the role of a protein can change through evolution across species, so the non-conservation of Clb3 may indicate a specificity of function in an organism, but not in another. For example, the budding yeast at usual growth rates appears to operate exclusively in the limit cycle domain, whereas the fission yeast operates mostly in a stable steady state domain. Thus, it seems likely that any molecule, e.g. Clb3 in budding yeast, is more important for some organisms than for others. Furthermore, the lack of a phenotype of a *clb3Δ* mutant suggests that the potentially less relevant genetic outcome for a gene deletion may hide a more sophisticated biochemical mechanism of regulation^47^.

Clb5, critical for the activation of DNA replication, is not essential in terms of survival because Clb2 can take over its role in *clb5*Δ cells^108^. However, Clb5 is considered in all computational models of the yeast cell cycle because of the experimental evidence of its role. Therefore, it is not surprising that also Clb3 is not an essential gene, with its function being taken over by Clb2 in *clb3*Δ cells, as mentioned above (see Pecani and Cross^109^ and details in Mondeel et al.^85^). Similarly, also Fkh2 is not an essential gene, with its function being partially taken over by Fkh1^43,112,113^, which may be involved in the regulation of Clb/Cdk1 activities^47^ although the molecular details of this mechanism are currently unknown.

Clb3 is lacking in existing computational models of the yeast cell cycle^91,92^, likely due to less explored functions as compared to those, well-known, of Clb5 and Clb2. However, although modulation of Clb3 activity is not required for mitotic exit^109^, its mitotic degradation is required for control of Start in G1 phase of the cell cycle. Strikingly, without mitotic destruction, Clb3 synthesized in the preceding cell cycle may directly activate Start, bypassing the requirement for the G1 (Cln) cyclins^109^. This evidence, together with the discovery of the role that Clb3 has in the formation of the waves of cyclins pattern^47^, has resulted in the inclusion of Clb3 in recent computational models of the yeast cell cycle^47,85^. Based on simulation results through a solid methodology^85^, Clb3 may be important for the robustness of autonomous limit cycle oscillations of three Clb/Cdk1 kinase complexes and of their stoichiometric inhibitor Sic1, where all three pairs of cyclins – including Clb3 – exhibit oscillations^22,23^.

After two decades of scientific gap regarding the role of Clb3, its critical role in the occurrence of the waves of cyclins pattern is now supported by both experimental^47^ and computational^85^ analyses, which have unraveled a molecular design that involves a Forkhead molecule^45^. Within this design, network structures incorporating elements of FFL + PFLs regulations (Fig. 2c) may be involved for the occurrence of Clb/Cdk1 oscillations, with a relevance for the NF-PFL motif for their autonomous pattern^86^.

Altogether, a novel design principle actuating the quantitative model of Cdk control for budding yeast is proposed that may rationalize the separation of the incompatible processes of genome duplication and cell division. This regulatory mode is such that a progressive activation of the Forkhead/Clb3 axis – possibly mediated by an increasing Clb/Cdk1-mediated multi-site phosphorylation of the Fkh2 transcription factor^102^ – controls the timing of cell cycle progression. While the present article was in press, a manuscript has appeared showing that the Fkh2 coactivator, Ndd1, undergoes multi-site phosphorylation by Clb3/Cdk1 and Clb2/Cdk1 – although to a different extent, with Clb2 being more effective than Clb3 – to regulate its degradation at cell cycle exit^114^. This evidence supports that multi-site phosphorylation by sequential activation of Clb/Cdk1 complexes modulates the timing of cell cycle dynamics^102^.

Given the evolutionary conservation of the cell cycle core machinery, this design principle of cellular proliferation that relies on cyclin/Cdk and transcription activities being interlocked may be envisioned in higher eukaryotes such as humans. Specifically, a core ‘*clock unit*’ incorporating this design has been recently proposed to be in place for both budding yeast and mammals^102^. It has a DRIVER (Cdk) operating its functions through multiple CLOCKS (mitotic Clb cyclins), with TIMERS (stoichiometric inhibitors of Clbs) determining whether and when the clocks are active, and CONTROLLERS (transcription factors) determining how quickly the clocks shall be active depending on external MODULATORS (e.g. epigenetic regulators)^102^. This ‘*clock unit*’ may coordinate temporal waves of cyclin/Cdk concentration/activity in the eukaryotic cell cycle, to keep the incompatible processes of genome duplication and cell division separated in time, thereby ensuring a robust and well-timed cell division.

## Data Availability

Data sharing not applicable to this article as no datasets were generated or analyzed during the current study.
